# Early statin use might reduce the hemorrhagic transformation among acute ischemic stroke patients with recanalization therapy: a retrospective cohort study

**DOI:** 10.3389/fphar.2025.1533905

**Published:** 2025-06-04

**Authors:** Boyan Pan, Jiaying Lan, Xiaojun Li, Haoxuan Chen, Luankun Weng, Haoyou Xu, Yuanqi Zhao, Min Zhao

**Affiliations:** ^1^ The Second Clinical College, Guangzhou University of Chinese Medicine, Guangzhou, China; ^2^ Zhongshan Hospital of Traditional Chinese Medicine Affiliated to Guangzhou University of Traditional Chinese Medicine, Zhongshan, China; ^3^ The Second Affiliated Hospital of Guangzhou University of Chinese Medicine, Guangdong Provincial Hospital of Chinese Medicine, Zhuhai, China; ^4^ The Second Affiliated Hospital of Guangzhou University of Chinese Medicine, Guangdong Provincial Hospital of Chinese Medicine, Guangzhou, China

**Keywords:** acute ischemic stroke, statins, hemorrhagic transformation, intravenous thrombolysis, endovascular treatment

## Abstract

**Objective:**

To evaluate the relationship between early statin administration and hemorrhagic transformation (HT) in patients with acute ischemic stroke (AIS) patients following recanalization therapy.

**Methods:**

This retrospective study included AIS patients who underwent recanalization therapy (intravenous thrombolysis, endovascular treatment, or a combination of both) and categorized them into two groups based on whether statins were administered within 24 h of recanalization therapy. The primary outcome was the occurrence of HT during hospitalization. Secondary outcomes included in-hospital mortality, favorable clinical outcomes (mRS 0–2) at discharge, and neurological improvement 7 ± 2 days post-stroke (defined as a reduction of ≥4 points in NIHSS from baseline).

**Results:**

A total of 266 AIS patients were analyzed, with 164 (61.7%) receiving statins within 24 h (24 h-statins group). The 24 h-statins group demonstrated a significantly lower risk of HT compared to the non-24 h-statins group (4.9% vs. 21.6%, p < 0.001). In-hospital mortality was also lower in the 24 h-statins group, although not statistically significant (4.9% vs. 10.8%, p = 0.076). Favorable clinical outcomes were more frequent in the 24 h-statins group than in the non-24 h-statins group (60.5% vs. 36.7%, p < 0.001). Furthermore, a greater proportion of patients in the 24 h-statins group showed neurological improvement (51.8% vs. 35.1%, p = 0.019). Adjusted multivariate analysis revealed that early statin use was independently associated with a reduced risk of HT (OR 0.16, 95% CI 0.06–0.49, p < 0.001), as well as a positive association with favorable clinical outcomes (OR 3.63, 95% CI 1.42–9.28, p = 0.007) and neurological improvement (OR 5.23, 95% CI 1.96–13.91, p < 0.001). Subgroup analysis indicated that among patients with elevated low-density lipoprotein (LDL) levels, early statin therapy was linked to a lower risk of HT (P for interaction = 0.018).

**Conclusion:**

Early statin administration within 24 h of recanalization therapy, in AIS patients was associated with reduced risk of HT and improved neurological outcomes. For patients with elevated LDL levels, early statin therapy may further decrease the risk of HT.

## 1 Introduction

Recanalization therapy, including intravenous thrombolysis (IVT) and endovascular treatment (EVT), represents the most effective treatment for acute ischemic stroke (AIS) in the ultra-early stage. This approach rapidly restores cerebral blood flow and helps to minimize further neuronal injury (Powers et al., 2019; Herpich and Rincon, 2020). However, recanalization therapy carries an increased risk of hemorrhagic transformation (HT), a serious and often fatal complication following AIS (Maïer et al., 2020; Tian et al., 2022), posing significant threats to patient survival and health.

Statins, or 3-hydroxy-3-methylglutaryl-coenzyme A (HMG-CoA) reductase inhibitors, are widely recognized for their beneficial role in preventing and managing cerebrovascular diseases (Amarenco et al., 2009; Åivo et al., 2023). Despite limited evidence, continuing or initiating statin therapy during the acute phase of ischemic stroke is considered reasonable (Powers et al., 2019). Most prior studies have suggested that statin use in AIS can improve patient prognosis (Hong and Lee, 2015; Åivo et al., 2023; Gao et al., 2024). However, controversy remains over whether statins increase the risk of HT in AIS patients, particularly among those undergoing recanalization therapy. While some studies indicated that combining statins with recanalization therapy does not elevate, and may even reduce the incidence of HT (Kang et al., 2015; Montaner et al., 2016; Tan et al., 2019). A meta-analysis reported that statins might raise the risk of HT (Hong and Lee, 2015). This increased risk may be linked to low lipid levels weakening endothelial integrity, potentially leading to vessel rupture (Lauer et al., 2015). These conflicting findings highlight the need for further research to evaluate the safety and efficacy of early statin use.

The objective of this study is to examine the relationship between early statin use and clinical outcomes, with a specific focus on HT in AIS patients following recanalization therapy.

## 2 Methods

This retrospective single-center cohort study was conducted with approval from the Ethics Committee of Guangdong Provincial Hospital of Chinese Medicine (YE 2020-069).

### 2.1 Study participants

A total of 279 consecutive AIS patients admitted to Guangdong Provincial Hospital of Chinese Medicine between 1 January 2018 and 30 April 2020 were included. All patients were registered with the National Stroke Center (http://sinosc.chinasdc.cn). The inclusion criteria were: (1) diagnosis of AIS; (2) receiving recanalization therapy, including IVT, EVT, or a combination of both. The exclusion criteria included: (1) missing critical data, such as the timing of IVT or EVT; (2) final diagnosis not being AIS, for example, transient ischemic attack (TIA), internal carotid artery dissection, *etc.*,; (3) length of hospitalization duration of less than 1 day; (4) HT during the process of recanalization therapy.

### 2.2 Definition of statin use

The 24 h-statins group comprised patients who received statins within 24 h following recanalization therapy. The non-24 h-statins group included patients who began statin therapy more than 24 h after recanalization therapy or those who did not receive statin therapy during hospitalization.

### 2.3 Outcome

The primary outcome was HT, assessed based on clinical and CT criteria by experienced neurologists. According to the European Cooperative Acute Stroke Study (ECASS) criteria, hemorrhagic infarction (HI) 1 was defined as small petechiae along the infarct margins, while HI2 was described as confluent petechiae within the infarcted area without space-occupying effects. Parenchymal hemorrhage (PH) 1 was defined as blood clots covering ≤30% of the infarcted area with minimal space-occupying effects, whereas PH2 referred to blood clots affecting >30% of the infarcted area with significant space-occupying effects (Hacke et al., 1998). Secondary outcomes included in-hospital mortality, favorable clinical outcomes (modified Rankin Scale score 0–2 at discharge) (Haggag and Hodgson, 2022), and neurological improvement (defined as an National Institute of Health stroke scale (NIHSS) score improvement of ≥4 points from baseline to 7 ± 2 days after recanalization therapy) (Agarwal et al., 2020).

### 2.4 Data collection

Data were collected using a standardized Case Report Form, including variables such as age, gender, baseline NIHSS scores, blood pressure, medical history, prior use of antiplatelet or anticoagulants, vascular occlusion site, type of recanalization therapy, the time of recanalization therapy, in-hospital drug therapies, and blood test results.

### 2.5 Statistical analyses

All statistical analyses were conducted using the Statistical Package for Social Sciences (SPSS) version 18.0 for Windows. Differences between the 24 h-statins group and the non-24 h-statins group were analyzed using the χ^2^ test, independent sample t-test, or Kruskal–Wallis test, as appropriate. Logistic regression models were utilized to examine the relationship between outcomes and statin use, calculating odds ratios (OR) with 95% confidence intervals (95% CI). In multivariate logistic regression model 1, adjustments were made for previously reported risk factors and all variables with p < 0.05 in univariate analyses. In model 2, adjustments included previously reported risk factors and variables with p < 0.1 in univariate analyses.

## 3 Results

### 3.1 Study population

**FIGURE 1:**
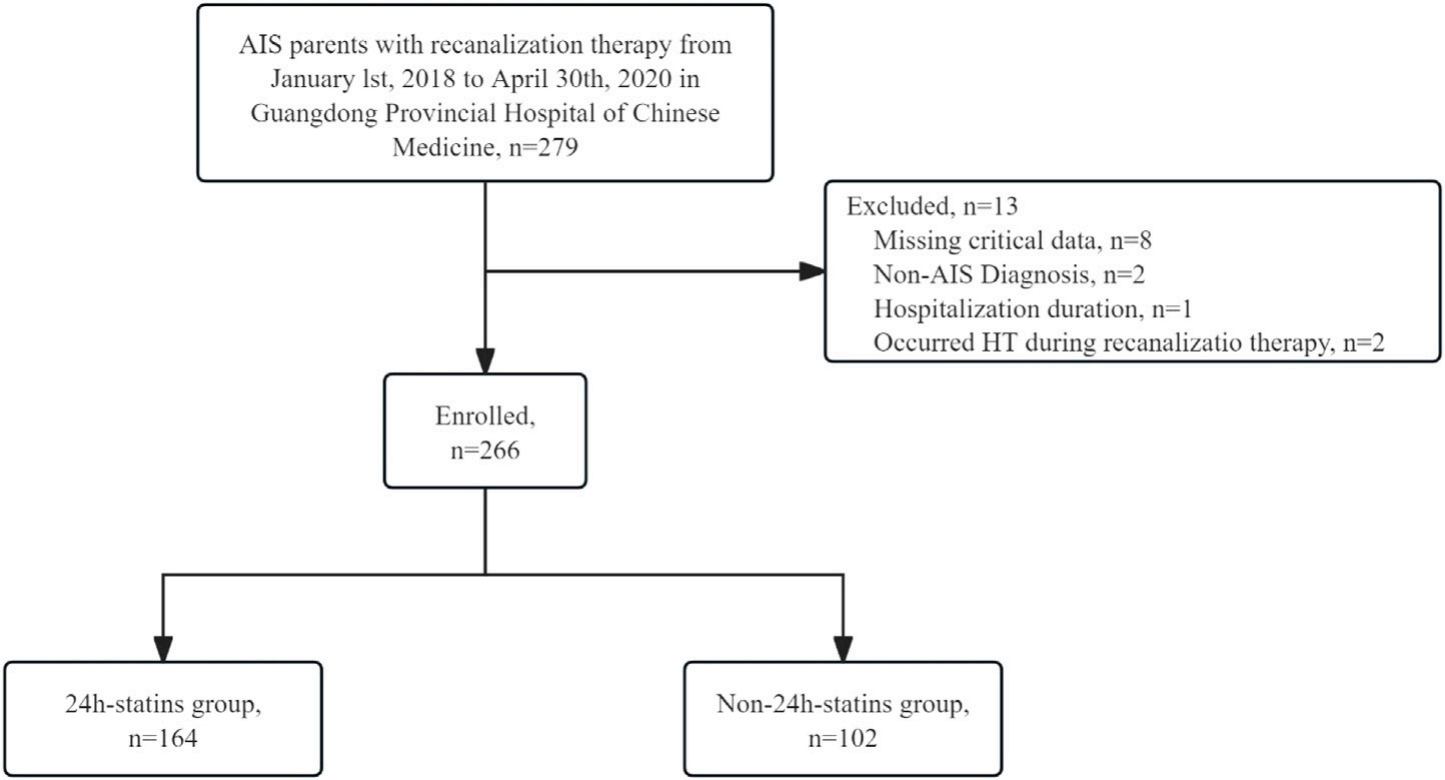
Study population AIS, acute ischemic stroke; HT = hemorrhagic transformation.

Between 1 January 2018 and 30 April 2020, 279 consecutive AIS patients treated with IVT or EVT were screened. A total of 13 patients were excluded, including 8 with missing critical data, 1 with a length of hospitalization duration less than 1 day, 2 with a final diagnosis other than AIS and two who experienced HT during recanalization therapy. Ultimately, 266 patients were included in the analysis. Among these, 110 (41.4%) patients received IVT, 111 (41.7%) underwent EVT, and 45 (16.9%) received a combination of IVT and EVT. Of the 266 patients, 164 (61.7%) were in the 24 h-statins group, while 102 (38.3%) were in the non-24 h-statins group (Figure 1).

### 3.2 Baseline characteristics

The non-24 h-statins group included more male patients (62.7% vs. 57.3%, p = 0.381) and had a higher mean age (68.2 ± 12.8 years vs. 66.7 ± 11.4 years, p = 0.142) compared to the 24 h-statins group. A significantly higher proportion of patients in the non-24 h-statins group had a history of coronary heart disease (CHD) (21.6% vs. 12.2%, p = 0.041). Additionally, more patients in the non-24 h-statins group underwent combined recanalization therapy (24.5% vs. 12.2%, p = 0.009). Other patient characteristics, including medical history (ischemic stroke, diabetes, hypertension, atrial fibrillation, valvular heart disease), prior use of antiplatelet agents (9.8% vs. 6.1%, p = 0.265), prior use of anticoagulants (7.8% vs. 4.3%, p = 0.219), baseline SBP (152.6 ± 27.3 mmHg vs. 149.2 ± 23.9 mmHg, p = 0.413), baseline NIHSS scores (12.1 ± 7.2 vs. 10.8 ± 7.1, p = 0.104), vascular occlusion site, and antiplatelet or anticoagulant use within 24 h, showed no significant differences between the two groups. However, the non-24 h-statins group had significantly higher fibrinogen (FIB) levels (3.5 ± 1.0 g/L vs. 3.1 ± 1.1 g/L, p = 0.001) and longer activated partial thromboplastin time (APTT) (32.2 ± 6.2 s vs. 28.9 ± 6.4 s, p < 0.001) than the 24 h-statins group. No significant differences were observed in other baseline laboratory values (Table 1).

**TABLE 1:** Clinical characteristics of the 24 h-statins group and the non-24 h-statins group.

Characteristics	Total n = 266	24 h-statins group, n = 164 (61.7%)	Non-24 h-statins group n = 102 (38.3%)	*P* value
Male, No. (%)	158 (59.4)	94 (57.3)	64 (62.7)	0.381
Age, mean (SD)	67.3 ± 12.0	66.7 ± 11.4	68.2 ± 12.8	0.142
Medical history				
Ischemic stroke, No. (%)	45 (16.9)	28 (17.1)	17 (16.7)	0.931
Diabetes, No. (%)	59 (22.2)	39 (23.8)	20 (19.6)	0.426
Hypertension, No. (%)	158 (59.4)	95 (57.9)	63 (61.8)	0.535
Coronary heart disease, No. (%)	42 (15.8)	20 (12.2)	22 (21.6)	0.041
Atrial fibrillation, No. (%)	58 (21.8)	30 (18.3)	28 (27.5)	0.079
Valvular heart disease, No. (%)	37 (13.9)	23 (14.0)	14 (13.7)	0.945
Prior use of antiplatelet, No. (%)	20 (7.5)	10 (6.1)	10 (9.8)	0.265
Prior use of anticoagulant, No. (%)	15 (5.6)	7 (4.3)	8 (7.8)	0.219
SBP, mean (SD), mmHg	150.5 ± 25.2	149.2 ± 23.9	152.6 ± 27.3	0.413
DBP mean (SD), mmHg	85.1 ± 14.5	85.2 ± 13.7	85.0 ± 15.8	0.747
Stroke severity				
Baseline NIHSS score, mean (SD)	11.3 ± 7.1	10.8 ± 7.1	12.1 ± 7.2	0.104
Vascular occlusion site				
Anterior circulation, No. (%)	163 (63.2)	99 (61.5)	64 (66.0)	0.469
Posterior circulation, No. (%)	68 (26.4)	42 (26.1)	26 (26.8)	0.899
Type of Recanalization therapy				
IVT, No. (%)	110 (41.4)	72 (43.9)	38 (37.3)	0.284
EVT, No. (%)	111 (41.7)	72 (43.9)	39 (38.2)	0.362
IVT + EVT, No. (%)	45 (16.9)	20 (12.2)	25 (24.5)	0.009
Process time, mean (SD)				
ONT, mean (SD), min	149.20 ± 57.91	148.75 ± 52.40	149.84 ± 65.34	0.913
OPT, mean (SD), min	412.19 ± 245.96	440.23 ± 264.72	371.68 ± 211.52	0.077
Antiplatelet use within 24h, No. (%)	111 (41.7)	74 (45.1)	37 (36.3)	0.155
Anticoagulant use within 24h, No. (%)	2 (0.8)	1 (0.6)	1 (1.0)	1.000
Blood test				
Hb, mean (SD), g/L	136.3 ± 19.4	136.2 ± 19.4	136.4 ± 19.5	0.754
PLT, mean (SD), $10^{9}$ /L	230.8 ± 62.5	228.7 ± 57.9	234.5 ± 70.0	0.750
INR, mean (SD)	0.990 ± 0.109	0.987 ± 0.111	0.995 ± 0.106	0.367
FIB, mean (SD), g/L	3.3 ± 1.1	3.1 ± 1.1	3.5 ± 1.0	0.001
APTT, mean (SD), s	30.1 ± 6.5	28.9 ± 6.4	32.2 ± 6.2	<0.001
LDL, mean (SD), mmol/L	2.9 ± 1.0	3.0 ± 1.0	2.9 ± 1.0	0.801
Glu, mean (SD), mmol/L	8.5 ± 3.7	8.5 ± 3.6	8.5 ± 3.9	0.676
UA, mean (SD), μmol/L	342.6 ± 110.1	342.2 ± 112.5	343.5 ± 106.3	0.742
HCY, mean (SD), μmol/L	11.7 ± 4.4	11.9 ± 4.6	11.6 ± 4.2	0.990

SBP, systolic blood pressure; DBP, diastolic blood pressure; NIHSS, national institute of health stroke scale; IVT, intravenous thrombolysis; EVT, endovascular treatment; ONT, Onset-to-Needle Time, OPT, Onset-to-Procedure Time, Hb, hemoglobin, PLT, platelet; INR, international normalized ratio; FIB, fibrinogen; APTT, activated partial thromboplastin time; LDL, low density lipoprotein, Glu, glucose, UA, uric acid; HCY, homocysteine.

### 3.3 Comparison of outcomes

The overall occurrence rate of HT was 11.3% (n = 30), with a significantly lower rate observed in the 24 h-statins group (4.9%) compared to the non-24 h-statins group (21.6%, p < 0.001). Among the 19 patients (7.1%) who died before discharge, 8 (4.9%) were in the 24 h-statins group, while 11 (10.8%) were in the non-24 h-statins group (p = 0.076). A total of 134 patients (51.5%) achieved favorable clinical outcomes at discharge, with a higher proportion in the 24 h-statins group compared to the non-24 h-statins group (60.5% vs. 36.7%, p < 0.001). Among 98 patients (45.8%) who demonstrated neurological improvement 7 ± 2 days post-recanalization therapy, 71 (51.8%) were in the 24 h-statins group, compared to 27 (35.1%) in the non-24 h-statins group (p = 0.019) (Table 2).

**TABLE 2:** Outcomes of the 24 h-statins group and the non-24 h statins group.

Outcome, no. (%)	Total	24 h-statins group (n = 164)	Non-24 h-statins group (n = 102)	*P* value	OR (95% CI)
Hemorrhagic transformation	30 (11.3)	8 (4.9)	22 (21.6)	<0.001	0.2 (0.1–0.4)
HI	10 (33.3)	5 (62.5)	5 (22.7)	0.051	5.7 (1.0, 32.4)
HI1	2 (6.7)	1 (12.5)	1 (4.5)	0.458	3.0 (0.2, 54.6)
HI2	8 (26.7)	4 (50.0)	4 (18.2)	0.094	4.5 (0.8, 26.1)
PH	20 (66.7)	3 (37.5)	17 (77.2)	0.051	0.2 (0.0, 1.0)
PH1	5 (16.7)	0 (0.0)	5 (22.7)	0.996	0.0 (0.0, Inf)
PH2	15 (50.0)	3 (37.5)	12 (54.5)	0.413	0.5 (0.1, 2.6)
In-hospital mortality	19 (7.1)	8 (4.9)	11 (10.8)	0.076	0.4 (0.2–1.1)
Favorable clinical outcome	134 (51.5)	98 (60.5)	36 (36.7)	<0.001	2.6 (1.6–4.4)
Neurologic improvement	98 (45.8)	71 (51.8)	27 (35.1)	0.019	2.0 (1.1–3.5)

CI, confidence interval; OR, odds ratio; HI, hemorrhagic infarction; PH, parenchymal hemorrhage.

### 3.4 Univariate analysis

Univariate analysis identified baseline NIHSS scores (OR 1.06, 95% CI 1.00–1.11) as being associated with an increased risk of HT, while IVT was associated with a lower risk of HT (OR 0.2, 95% CI 0.1–0.7). Baseline NIHSS scores (OR 1.1, 95% CI 1.0–1.2), EVT (OR 3.3, 95% CI 1.2–9.0), and female sex (male: OR 0.4, 95% CI 0.1–1.0) were associated with higher mortality. Regarding favorable clinical outcomes, baseline NIHSS scores (OR 0.8, 95% CI 0.8–0.9), hypertension (OR 0.6, 95% CI 0.3–0.9), CHD (OR 0.4, 95% CI 0.2–0.9), prior antiplatelet use (OR 0.3, 95% CI 0.1–1.0), anterior circulation stenosis (OR 0.4, 95% CI 0.2–0.6), posterior circulation stenosis (OR 0.5, 95% CI 0.3–0.9), EVT (OR 0.3, 95% CI 0.2–0.5), FIB levels (OR 0.7, 95% CI 0.5–0.9), and glucose levels (OR 0.9, 95% CI 0.8–1.0) were identified as risk factors. IVT (OR 3.9, 95% CI 2.3–6.6) emerged as a protective factor. For neurological improvement, higher age (OR 1.02, 95% CI 1.00–1.05), a medical history of atrial fibrillation (AF) (OR 2.0, 95% CI 1.1–3.8), higher baseline NIHSS scores (OR 1.15, 95% CI 1.09–1.20), and anterior circulation stenosis (OR 2.0, 95% CI 1.1–3.5) were associated with greater improvement. Patients who received IVT (OR 0.5, 95% CI 0.3–0.9) were associated with poorer improvement (Table 3).

**TABLE 3:** Univariate analysis of outcomes.

Characteristics	Hemorrhagic transformation		In-hospital mortality		Favorable outcome		Neurologic improvement	
	OR (95% CI)	*P* value	OR (95% CI)	*P* value	OR (95% CI)	*P* value	OR (95% CI)	*P* value
Demographic data								
Male	0.6 (0.3–1.2)	0.135	0.4 (0.1–1.0)	0.044	1.3 (0.8–2.2)	0.243	0.8 (0.5–1.4)	0.477
Age	1.00 (0.97–1.03)	0.868	1.03 (0.98–1.07)	0.227	0.98 (0.96–1.00)	0.055	1.02 (1.00–1.05)	0.045
Medical history								
Ischemic stroke	1.3 (0.5–3.3)	0.633	0.9 (0.3–3.3)	0.892	0.7 (0.3–1.3)	0.221	1.7 (0.8–3.5)	0.144
Diabetes	1.6 (0.7–3.7)	0.277	2.2 (0.8–5.8)	0.118	0.7 (0.4–1.3)	0.245	0.7 (0.4–1.3)	0.263
Hypertension	1.4 (0.6–3.2)	0.391	1.5 (0.6–4.1)	0.409	0.6 (0.3–0.9)	0.026	1.5 (0.9–2.6)	0.148
Coronary heart disease	1.1 (0.4–3.0)	0.889	1.0 (0.3–3.6)	1.000	0.4 (0.2–0.9)	0.017	1.4 (0.7–3.1)	0.373
Atrial fibrillation	1.4 (0.6–3.2)	0.495	1.7 (0.6–4.8)	0.289	0.7 (0.4–1.3)	0.245	2.0 (1.1–3.8)	0.034
Valvular heart disease	1.7 (0.6–4.4)	0.310	1.2 (0.3–4.2)	0.806	0.7 (0.4–1.5)	0.360	1.4 (0.7–2.9)	0.363
Prior use of antiplatelet	0.9 (0.2–3.9)	0.851	1.5 (0.3–7.0)	0.608	0.3 (0.1–1.0)	0.045	1.2 (0.4–3.5)	0.744
Prior use of anticoagulants	3.1 (0.9–10.6)	0.064	0.9 (0.1–7.4)	0.941	1.1 (0.4–3.1)	0.886	1.0 (0.3–3.1)	0.979
Blood pressure								
SBP	1.00 (0.99–1.02)	0.813	1.01 (0.99–1.03)	0.273	0.99 (0.98–1.00)	0.061	1.00 (0.99–1.01)	0.688
DBP	1.00 (0.97–1.02)	0.813	1.01 (0.98–1.04)	0.434	0.99 (0.98–1.01)	0.453	1.00 (0.99–1.02)	0.686
Stroke severity								
Baseline NIHSS score	1.06 (1.00, 1.11)	0.035	1.1 (1.0–1.2)	0.003	0.8 (0.8–0.9)	<0.001	1.15 (1.09, 1.20)	<0.001
Vascular occlusion site								
Anterior circulation	1.9 (0.8–4.6)	0.175	2.1 (0.7–6.7)	0.192	0.4 (0.2–0.6)	<0.001	2.0 (1.1–3.5)	0.022
Posterior circulation	0.6 (0.2–1.6)	0.285	1.9 (0.7–5.0)	0.217	0.5 (0.3–0.9)	0.020	1.0 (0.5–1.8)	0.889
Type of recanalization therapy								
IVT	0.2 (0.1–0.7)	0.006	0.4 (0.1–1.1)	0.073	3.9 (2.3–6.6)	<0.001	0.5 (0.3–0.9)	0.014
EVT	2.0 (0.9–4.3)	0.082	3.3 (1.2–9.0)	0.020	0.3 (0.2–0.5)	<0.001	1.6 (0.9–2.7)	0.105
IVT + EVT	2.0 (0.8–4.7)	0.136	0.6 (0.1–2.5)	0.447	0.7 (0.4–1.4)	0.377	1.5 (0.7–3.1)	0.272
Process time								
ONT	0.99 (0.97–1.00)	0.245	1.00 (0.98–1.04)	0.663	0.99 (0.97–1.00)	0.157	1.01 (1.00–1.03)	0.154
OPT	1.00 (0.98–1.01)	0.670	1.00 (0.99–1.02)	0.675	1.00 (0.99–1.01)	0.673	1.00 (0.99–1.01)	0.350
Antiplatelet use within 24 h	0.8 (0.4–1.7)	0.551	1.0 (0.4–2.6)	0.972	0.7 (0.4–1.1)	0.121	1.0 (0.6–1.7)	0.889
Anticoagulant use within 24 h	0.0 (0.0, Inf)	0.990	0.0 (0.0, Inf)	0.990	0.9 (0.1–15.2)	0.965	1.2 (0.1–19.2)	0.905
Blood test								
Hb	0.99 (0.97–1.01)	0.235	0.99 (0.97–1.01)	0.338	1.01 (0.99–1.02)	0.397	0.99 (0.98–1.01)	0.341
PLT	0.997 (0.991, 1.004)	0.455	1.00 (0.99–1.01)	0.874	0.998 (0.994, 1.002)	0.277	0.995 (0.990, 1.001)	0.081
INR	18.2 (0.8–430.5)	0.072	6.5 (0.1–312.3)	0.344	0.8 (0.1–7.8)	0.834	7.6 (0.5–109.9)	0.134
FIB	1.2 (0.8–1.7)	0.309	1.0 (0.6–1.6)	0.921	0.7 (0.5–0.9)	0.003	0.9 (0.7–1.2)	0.609
APTT	1.04 (0.99, 1.10)	0.144	1.0 (0.9–1.1)	0.991	1.01 (0.97, 1.05)	0.632	1.03 (0.98, 1.07)	0.219
LDL	0.7 (0.4–1.2)	0.183	0.6 (0.3–1.2)	0.128	1.3 (1.0–1.7)	0.090	0.8 (0.6–1.1)	0.249
Glu	1.0 (0.9–1.1)	0.395	1.1 (1.0–1.2)	0.063	0.9 (0.8–1.0)	0.023	0.9 (0.8–1.0)	0.004
UA	1.001 (0.996, 1.006)	0.800	1.00 (0.99–1.01)	0.832	0.999 (0.996–1.002)	0.377	1.001 (0.998–1.004)	0.605
HCY	1.0 (0.8–1.2)	0.847	1.1 (1.0–1.3)	0.130	0.94 (0.86, 1.03)	0.217	1.0 (0.9–1.1)	0.403

CI, confidence interval; OR, odds ratio; NA, not applicable; SBP, systolic blood pressure; DBP, diastolic blood pressure; NIHSS, national institute of health stroke scale; IVT, intravenous thrombolysis; EVT, endovascular treatment; ONT, Onset-to-Needle Time, OPT, Onset-to-Procedure Time, Hb, hemoglobin, PLT, platelet; INR, international normalized ratio; FIB, fibrinogen; APTT, activated partial thromboplastin time; LDL, low density lipoprotein, Glu, glucose, UA, uric acid; HCY, homocysteine.

### 3.5 Multivariate analysis

After adjusting for influencing factors, multivariate analysis revealed a negative association between the 24 h-statins group and the risk of HT (OR 0.16, 95% CI 0.06–0.49, p < 0.001). There was no significant association observed between statin use and in-hospital mortality (OR 0.43, 95% CI 0.13–1.43, p = 0.172). The 24 h-statins group demonstrated a positive correlation with favorable clinical outcomes (OR 3.63, 95% CI 1.42–9.28, p = 0.007) and neurological improvement (OR 5.23, 95% CI 1.96–13.91, p < 0.001) (Table 4).

**TABLE 4:** Multivariate analysis of outcomes.

Outcome	Model 1			Model 2		
	No. (%)	OR (95% CI)	*P* value	No. (%)	OR (95% CI)	*P* value
Hemorrhagic transformation	247 (92.9)	0.16 (0.06, 0.49)	<0.001	247 (92.9)	0.19 (0.08, 0.45)	<0.001
In-hospital mortality	247 (92.9)	0.38 (0.12, 1.17)	0.910	238 (89.5)	0.43 (0.13, 1.43)	0.172
Favorable clinical outcome	226 (85.0)	2.88 (1.30, 6.38)	0.009	183 (68.8)	3.63 (1.42, 9.28)	0.007
Neurologic improvement	194 (73.0)	5.23 (1.96, 13.92)	0.001	187 (70.3)	5.23 (1.96, 13.91)	<0.001

CI, confidence interval; OR, odds ratio.

Model 1: adjust for gender, age, SBP, prior use of antithrombotics, baseline NIHSS, coronary heart disease, IVT + EVT, FIB, level; APTT, level and other variables with P < 0.05 in the univariate analysis.

Model 2: adjust for gender, age, SBP, prior use of antithrombotics, baseline NIHSS, coronary heart disease, IVT + EVT, FIB, level; APTT, level and other variables with P < 0.1 in the univariate analysis.

### 3.6 Subgroup analysis

Further subgroup analysis was conducted on factors of concern related to hemorrhage, specifically HT outcomes. High low-density lipoprotein (LDL) was defined as an LDL level >3.37 mmol/L, based on laboratory reference values. No significant interaction was found between the 24 h-statins group and CHD, type of recanalization therapy, or antiplatelet use. However, subgroup analysis indicated that in patients with high LDL levels, early statin use was associated with a reduced risk of HT (P for interaction = 0.018) (Table 5).

**TABLE 5:** Subgroup analysis of hemorrhagic transformation.

Characteristics	24 h-statins group	Non-24 h-statins group	Crude model		P For interaction	Model 1		P For interaction	Model 2		P For interaction
			OR (95% CI)	P value		OR (95% CI)	P value		OR (95% CI)	P value	
CHD, No. (%)											
Yes	20 (12.20)	22 (21.57)	0.2 (0.0, 1.4)	0.107	0.817	0.0 (0.0, Inf)	1.0000	0.217	0.0 (0.0, Inf)	1.0000	0.223
No	144 (87.80)	80 (78.43)	0.2 (0.1, 0.4)	<0.001		0.2 (0.1, 0.6)	0.0046		0.2 (0.0, 0.6)	0.0036	
IVT, No. (%)											
Yes	72 (43.90)	38 (37.25)	0.1 (0.0, 0.3)	<0.001	0.515	0.1 (0.0, 0.5)	0.0083	0.278	0.4 (0.1, 2.8)	0.3498	0.193
No	92 (56.10)	64 (62.75)	0.2 (0.1, 0.4)	<0.001		0.1 (0.0, 0.5)	0.0018		0.1 (0.0, 0.4)	0.0010	
EVT, No. (%)											
Yes	72 (43.90)	39 (38.24)	0.4 (0.1, 1.2)	0.097	0.340	1.0 (1.0, 1.0)	NA	0.361	0.1 (0.0, 0.5)	0.0045	0.357
No	92 (56.10)	63 (61.76)	0.3 (0.1, 0.9)	0.038		0.2 (0.1, 1.0)	0.0535		0.2 (0.0, 1.0)	0.0492	
IVT + EVT, No. (%)											
Yes	20 (12.20)	25 (24.51)	0.4 (0.1, 2.0)	0.281	0.470	0.2 (0.0, 1.4)	0.1097	0.922	0.1 (0.0, 1.1)	0.0603	0.717
No	144 (87.80)	77 (75.49)	0.2 (0.1, 0.4)	<0.001		0.2 (0.0, 0.6)	0.0046		0.2 (0.0, 0.5)	0.0041	
Antiplatelet use within 24h, No. (%)											
Yes	74 (45.12)	37 (36.27)	0.1 (0.0, 0.5)	0.008	0.024	0.0 (0.0, 0.3)	0.0027	0.065	0.0 (0.0, 0.3)	0.0033	0.099
No	90 (54.88)	65 (63.73)	0.4 (0.1, 1.0)	0.052		0.3 (0.1, 1.0)	0.0528		0.3 (0.1, 0.9)	0.0381	
LDL-high (>3.37 mmol/L), No. (%)											
Yes	42 (31.1)	17 (21.8)	0.0 (0.0, Inf)	0.987	0.041	0.0 (0.0, Inf)	1.0000	0.026	0.0 (0.0, Inf)	1.0000	0.018
No	93 (68.89)	61 (78.21)	0.4 (0.2, 1.1)	0.089		0.5 (0.1, 2.0)	0.3284		0.5 (0.1, 2.1)	0.3638	

CI, confidence interval; OR, odds ratio; NA, not applicable; IVT, intravenous thrombolysis; EVT, endovascular treatment; CHD, coronary heart disease.

Model 1: adjust for gender, age, SBP, prior use of antithrombotics, baseline NIHSS, coronary heart disease, IVT + EVT, FIB, level; APTT, level and other variables with P < 0.05 in the univariate analysis.

Model 2: adjust for gender, age, SBP, prior use of antithrombotics, baseline NIHSS, coronary heart disease, IVT + EVT, FIB, level; APTT, level and other variables with P < 0.1 in the univariate analysis.

## 4 Discussion

As we all know, statins are a cornerstone in the treatment of ischemic stroke and are widely used during the acute phase, recovery phase, and sequelae phase. The majority of the studies focuses on the efficacy of statins during the acute and recovery phases of ischemic stroke, while studies on the ultra-early phase are very limited. Currently, an increasing number of patients are receiving IVT, EVT, and bridging therapy. For these patients, it remains unclear whether early statin therapy can improve outcomes or increase the risk of intracranial hemorrhage. This uncertainty constitutes the primary objective and significance of the present study.

### 4.1 The association of statin use and the risk of HT

This study retrospectively analyzed 266 AIS patients treated with recanalization therapy and found that statin administration within 24 h after IVT or EVT was associated with a lower risk of HT, favorable clinical outcomes at discharge, and short-term neurological improvement, compared to patients in the non-24 h-statins group.

Since the rapid pro-fibrinolytic and anti-thrombotic mechanisms of statin treatment started may be protective during the acute phase (German and Liao, 2023). There remains a debate regarding whether statins treatment in the acute phase increases the risk of HT. Manuel Cappellari et al. (Cappellari et al., 2011) reported that AIS patients who received IVT and initiated statin use before hospitalization, continuing within 24 h of stroke onset, had a higher risk of symptomatic intracerebral hemorrhage. In contrast, a retrospective study (Gong et al., 2024) involving 510 cardioembolic stroke patients treated with EVT demonstrated that statin use within 7 days of the acute phase was associated with better clinical outcomes and a lower risk of symptomatic intracerebral hemorrhage compared to those who did not use statins. Similarly, the STARS trial (Montaner et al., 2016) confirmed that the combination of statins and IVT is safe and associated with a low incidence of HT. Different with the previous studies, our study not only encompasses patients with cardioembolic stroke but also investigated the effects of initiating statins within 24 h on HT and other clinical outcomes. Evidence from animal models also supports these results. In experiments involving early statin use during the acute phase of middle cerebral artery occlusion (MCAO) after IVT, statins did not increase and even reduced the risk of HT (Wu et al., 2019). Additionally, lovastatin use in intracerebral hemorrhage rat models did not exacerbate hemorrhage but instead inhibited further brain damage (Deng et al., 2023).

We hypothesize that statins reduce the risk of HT due to their pleiotropic effects. Statins exhibit anti-neuroinflammatory properties, improve endothelial dysfunction, and promote angiogenesis and tight vascular junctions (Reuter et al., 2015; Ma et al., 2021; Yang et al., 2021). These mechanisms may help reduce and repair damage to the blood-brain barrier caused by cerebral ischemia or reperfusion therapy. Further prospective cohort studies and interventional trials were needed to verify its efficacy.

### 4.2 The timepoint of statin use and prognosis

Current guidelines recommend initiating statin therapy for AIS patients during hospitalization (Powers et al., 2019). However, they do not provide specific recommendations regarding the optimal timing for statin initiation in AIS patients following recanalization therapy. Many studies have confirmed that initiating statin therapy within 72 h of recanalization may improve neurological outcomes. Jihoon Kang et al. (Kang et al., 2015) conducted a retrospective analysis of AIS patients undergoing recanalization therapy, including IVT and intra-arterial treatment. Their findings indicated that initiating statins on the first day after recanalization therapy was more effective in improving functional outcomes and reducing the risk of HT than starting statins on the second or third day. Similarly, a prospective study (Cappellari et al., 2013) of 2,072 AIS patients revealed that statin use within 72 h after IVT was associated with neurological improvement at 7 days, favorable functional outcomes at 3 months, and lower 90-day mortality. Statin use within 24 h demonstrated even greater efficacy. The ASCENT study (Havlíček et al., 2024) further reported that statin administration within 24 h after EVT was a significant predictor of favorable clinical outcomes at 3 months, with no evidence suggesting an association between statin use and symptomatic intracerebral hemorrhage. Our study further demonstrates that the use of statins within 24 h after revascularization therapy is safe and is associated with more favorable neurological outcomes. However, as these studies are primarily observational, further randomized controlled trials are required to verify it.

### 4.3 The interaction between LDL level and the risk of HT

Our subgroup analysis revealed that the effect of statins in reducing the risk of HT was more pronounced in patients with high LDL levels (>3.37 mmol/L). As is well known, following AIS, the damaged neurons release signals that activate the immune response, including the activation of glial cells and the release of various inflammatory mediators. These neuroinflammatory processes can further accelerate the disruption of the blood–brain barrier, exacerbate oxidative stress, and thereby cause secondary brain injury, such as the occurrence of HT (Rosell et al., 2008; Shi et al., 2019). This process is more intense in patients who have undergone reperfusion therapy (Khatri et al., 2012; Sun et al., 2018). Elevated plasma LDL levels can further stimulate the release of pro-inflammatory cytokines, accelerating the inflammatory response (Uno et al., 2005). Animal experimental studies have confirmed that dyslipidemia, characterized by high levels of LDL, can exacerbate brain injury in rats subjected to MCAO and reperfusion by inducing the formation of reactive oxygen species and promoting oxidative stress and inflammatory responses (Cao et al., 2015). Statins can mitigate brain injury induced by elevated LDL levels through their ability to reduce plasma LDL concentrations. In addition to their lipid-lowering effects, statins themselves also have anti-inflammatory and antioxidant properties (German and Liao, 2023). Therefore, in patients with high LDL levels, the use of statins can not only alleviate the enhanced inflammatory response caused by high LDL but also mitigate the adverse effects caused by cerebral ischemia/reperfusion. Hence, the effect of statins in reducing the risk of HT might be more pronounced in patients with high LDL levels (>3.37 mmol/L).

### 4.4 Limitations

There are limited studies exploring the relationship between early statin use and the prognosis of AIS patients undergoing EVT. Our findings suggest that statin use within 24 h of recanalization therapy is associated with a reduced risk of HT. However, several limitations must be acknowledged. First, as a single-center, retrospective study based on real-world data without long-term outcome data, such as 90-day mRS and mortality and the results should be interpreted with caution. Prospective studies are needed to further validate our findings. Secondly, this study defined statin use based solely on whether it was administered within 24 h, without considering factors such as the type of statin, dosage, or duration of use. These variables may significantly influence the effects of statins, and future research should aim to refine the definition of statin use by incorporating these critical aspects.

## 5 Conclusion

Our study demonstrated that in AIS patients undergoing recanalization therapy, statin use within 24 h was associated with a reduced risk of HT and improved neurological function. Subgroup analysis further indicated that in patients with elevated LDL levels, early statin use was linked to a lower risk of HT.

## Data Availability

The original contributions presented in the study are included in the article/supplementary material, further inquiries can be directed to the corresponding authors.
